# Detailed Analysis of Variants in FTO in Association with Body Composition in a Cohort of 70-Year-Olds Suggests a Weakened Effect among Elderly

**DOI:** 10.1371/journal.pone.0020158

**Published:** 2011-05-27

**Authors:** Josefin A. Jacobsson, Markus Sällman Almén, Christian Benedict, Lilia A. Hedberg, Karl Michaëlsson, Samantha Brooks, Joel Kullberg, Tomas Axelsson, Lars Johansson, Håkan Ahlström, Robert Fredriksson, Lars Lind, Helgi B. Schiöth

**Affiliations:** 1 Department of Neuroscience, Functional Pharmacology, Uppsala University, Uppsala, Sweden; 2 Science for Life Laboratory, Royal Institute of Technology (KTH), School of Biotechnology, Solna, Sweden; 3 Department of Surgical Sciences, Uppsala University, Uppsala, Sweden; 4 Department of Medical Sciences, Uppsala University, Uppsala, Sweden; 5 Department of Oncology, Radiology and Clinical Immunology, Uppsala University, Uppsala, Sweden; 6 AstraZeneca R&D Mölndal, Mölndal, Sweden; University of Tor Vergata, Italy

## Abstract

**Background:**

The rs9939609 single-nucleotide polymorphism (SNP) in the fat mass and obesity (FTO) gene has previously been associated with higher BMI levels in children and young adults. In contrast, this association was not found in elderly men. BMI is a measure of overweight in relation to the individuals' height, but offers no insight into the regional body fat composition or distribution.

**Objective:**

To examine whether the *FTO* gene is associated with overweight and body composition-related phenotypes rather than BMI, we measured waist circumference, total fat mass, trunk fat mass, leg fat mass, visceral and subcutaneous adipose tissue, and daily energy intake in 985 humans (493 women) at the age of 70 years. In total, 733 SNPs located in the *FTO* gene were genotyped in order to examine whether rs9939609 alone or the other SNPs, or their combinations, are linked to obesity-related measures in elderly humans.

**Design:**

Cross-sectional analysis of the Prospective Investigation of the Vasculature in Uppsala Seniors (PIVUS) cohort.

**Results:**

Neither a single SNP, such as rs9939609, nor a SNP combination was significantly linked to overweight, body composition-related measures, or daily energy intake in elderly humans. Of note, these observations hold both among men and women.

**Conclusions:**

Due to the diversity of measurements included in the study, our findings strengthen the view that the effect of *FTO* on body composition appears to be less profound in later life compared to younger ages and that this is seemingly independent of gender.

## Introduction

Genome-wide association studies have provided evidence for a relationship between prevalent variants in the fat mass and obesity associated (FTO) gene and obesity [1], [2], [3]. Interestingly, this association is most commonly seen in cohorts of children and young adults carrying the A-allele of the single nucleotide polymorphism (SNP), rs9939609, in the *FTO* gene [4], [5], [6], [7], [8], [9], [10], [11]. This genetic variant has been shown to be linked to increased BMI [12] and higher level of total body adiposity [13]. Furthermore, carriers of the rs9939609 polymorphism show more frequent selection of energy-dense, palatable foods [14] and are less sensitive to satiety cues [15]. In a proof-of-principle study, we have previously examined whether *FTO* is also associated with BMI in a large, homogenous sample of elderly men [16]. However, contrary to observations in children and young adults, we did not detect a significant influence of the *FTO* rs9939609 genotype on BMI levels in this elderly population. Although BMI is often used as surrogate measurement of adiposity, it has its limitations inasmuch as, starting in midlife, the ratio of fat mass to height increases with age [17]. Thus, it may be that a potential association between the *FTO* gene and body weight in elderly men was biased by the measurement itself. A further shortcoming of the previous study was that only men were included.

To examine whether *FTO* variants are associated with body composition rather than with the BMI level in elderly humans, we determined the *FTO* genotype of rs9939609 and an additional 732 SNPs covering all introns and exons of the gene. We also took extensive measurements of body composition, which included visceral and subcutaneous adipose tissue, total fat mass, trunk fat mass, leg fat mass and waist circumference in men and women at the age of 70 years. In addition, total dietary energy intake was obtained by seven-day food records.

Besides the *FTO* rs9939609 polymorphism, other common variants in this gene have also been associated with anthropometric measures and food intake behavior [18], [19], [20]. Intriguingly, SNPs in regions beyond the first intron have also been associated with obesity-related traits, indicating that specific regions in the very large *FTO* gene spanning more than 400 kb may be differentially associated with obesity-related phenotypes [21], [22]. Thus, we also performed a *FTO* gene-wide haplotype analysis on these 733 SNPs, providing greater power [23], [24] compared with evaluation of single markers, which adds to the understanding of the association between the overall *FTO* SNP linkage map with body fat composition.

## Materials and Methods

### Subjects

492 men and 493 women at the age of 70 years were recruited from a population based cohort study named the Prospective Investigation of the Vasculature in Uppsala Seniors (PIVUS) [25]. The study was conducted in Uppsala, Sweden, and was approved by the Ethics Committee of the University of Uppsala and all participants gave written informed consent.

### Anthropometrics measurements

Descriptive characteristics of the participants' age, height, weight, BMI, and body fat are presented in Table 1. In order to calculate the BMI (kg/m^2^), height and weight were measured in all participants to the nearest 0.5 cm and 0.5 kg, respectively. 38% of the participants had a BMI less than 25 kg/m^2^ whereas the remaining 610 participants had a BMI greater than 25 kg/m^2^ and were therefore considered overweight. Waist circumference was measured in all subjects in a supine position at the iliac crest. In order to quantify the visceral and subcutaneous adipose tissue compartments, 278 subjects (133 women, 145 men) were measured at the L4–L5 intervertebral level using magnetic resonance imaging (MRI; detailed description see [26]). Afterwards, both adipose tissues were quantified by converting the numbers of pixels into cm^2^. Total body fat mass, trunk fat mass and leg fat mass were estimated in a subsample of 860 subjects (443 women, 417 men) using Dual-energy X-ray absorptiometry (DXA; Lunar Prodigy, Lunar corp., Madison, WI, USA). By triple measurements in 15 subjects, the precision error of the DXA measurements in our laboratory has been calculated. Total fat mass had a precision error of 1.5%.

**Table 1:** Descriptive characteristics of the subjects included in the study.

	N	All	Male	Female
**Age**	985	70.2 (0.2)	70.3 (0.2)	70.2 (0.2)
**Height (cm)**	985	168.9 (9.1)	175.8 (6.5)	162.1 (5.6)
**Weight (kg)**	985	77.4 (14.5)	83.5 (13.0)	71.2 (13.2)
**BMI**	985	27.1 (4.3)	27.1 (4.9)	27.0 (3.7)
**Waist circumference (cm)**	974	100.8 (8.1)	100.2 (6.7)	101.3 (9.3)
**Total fat mass by DXA (kg)**	860	25.7 (9.0)	23.5 (8.3)	27.7 (9.3)
**Leg fat mass by DXA (kg)**	860	8.1 (3.5)	6.3 (2.6)	9.7 (3.4)
**Trunk fat mass by DXA (kg)**	860	14.1 (10.7)	14.2 (5.1)	13.9 (5.0)
**VAT (cm2)**	278	107.6 (58.2)	120.0 (63.2)	94.1 (48.9)
**SAT (cm2)**	278	224.8 (102.9)	189.2 (80.7)	263.6 (110.5)
**Energy intake (kJ)**	834	7903.3 (2119.5)	7211.0 (1750.0)	8592.0 (2230.0)

Values are geometric means ± SD. Abbreviation: BMI, body mass index, DXA, Dual-energy X-ray absorptiometry, VAT, visceral adipose tissue, SAT, subcutaneous adipose tissue.

### Seven-day food records

The total daily energy intake was measured in kJ and was calculated by means of a seven-day food and beverage intake diary in a subsample of 834 volunteers (416 women, 418 men). The participants used a pre-coded food diary after instructions from a dietician, used [27] and validated [28] previously. Information about energy and nutrient contents of foods and beverages was derived from a database provided by the Swedish National Food Administration and includes in total 1,500 food items, drinks, and recipes.

### Genotyping and linkage disequilibrium analysis

Altogether, a number of 1332 SNPs located in the *FTO* gene (chromosome 16) were genotyped in 985 subjects as a part of a custom Illumina iSelect genotyping array. All SNPs were successfully genotyped with an average call rate of 99.9%. 599 SNPs were excluded due to a minor allele frequency (MAF) less than 0.01, whereas 733 SNPs with a MAF >0.01 were considered eligible. According to the 1000 Genomes Project (browser.1000genomes.org), 37 variants were named after their chromosomal position (Chr16: position)(www.ncbi.nlm.nih.gov). Testing for Hardy-Weinberg equilibrium (using a χ^2^-test, 1 d.f) revealed that none of the SNPs deviated from expected genotype proportion. Haploview [29] was used to construct haplotype blocks according to confidence intervals by Gabriel et al [30] as well as for graphical representation of the LD structure indicated as r^2^. Comparison between the LD patterns generated by HapMap and the PIVUS data was conducted using Haploview and the HapMap data version 3, release 27 and CEU+TSI as analysis panel.

### Statistical analysis

The association between single markers and haplotypes in the *FTO* gene with BMI, waist circumference, VAT, SAT, total fat mass, trunk fat mass, leg fat mass, and energy intake was tested with linear regression assuming an additive model. For this, the statistical software PLINK (http://pngu.mgh.harvard.edu/purcell/plink/) was used. In case of the dichotomized variable overweight, based on subjects categorized as overweight (BMI ≥25 kg/m^2^) and normal weight (BMI <25 kg/m^2^), a logistic regression model was applied. Quantitative skewed variables were normalized by log-transformation before analysis. All analyses were adjusted for gender. Interaction of *FTO* variants with gender on the obesity-related traits were analyzed by introducing a product interaction term in the model. In order to correct for multiple comparisons Bonferroni correction was applied. In addition, a 10 000 permutation test was conducted with the max(T) procedure in PLINK in order to establish gene-wide empirical p-values. A p-value less than 8.5×10^−6^ was considered statistically significant. Graphical representation of the –log10 p-values was performed with Graphpad Prism version 5.02 (GraphPad Software, San Diego, USA).

### Power analysis

Power calculations were carried out with the CaTS power calculator (www.sph.umich.edu/csg/abecasis/CaTS)[31] and Power and Sample Size Calculation (biostat.mc.vanderbilt.edu/wiki/Main/PowerSampleSize)[32]. For the case/control comparisons, power was estimated using a disease model as reported for rs9939609 (MAF 0.46) by Frayling et al. [1]. For SNPs with MAF <0.05, we had 10% power to detect association with overweight. For SNPs with MAF >0.10 and MAF >0.30 we had ≥50% and ≥80% power to detect association with overweight. For the quantitative phenotypes we had 80 % power to detect the following changes per allele; BMI 0.3, waist circumference 1.0 cm, energy intake 202 kj, VAT 9.6 cm^2^, SAT 17 cm^2^, total fat mass 0.8 kg, trunk fat mass 0.4 kg and leg fat mass 0.3 kg.

## Results

### Associations between single nucleotide polymorphisms in the fat mass and obesity associated (FTO) gene and anthropometric measurements as well as energy intake

**Figure 1:**
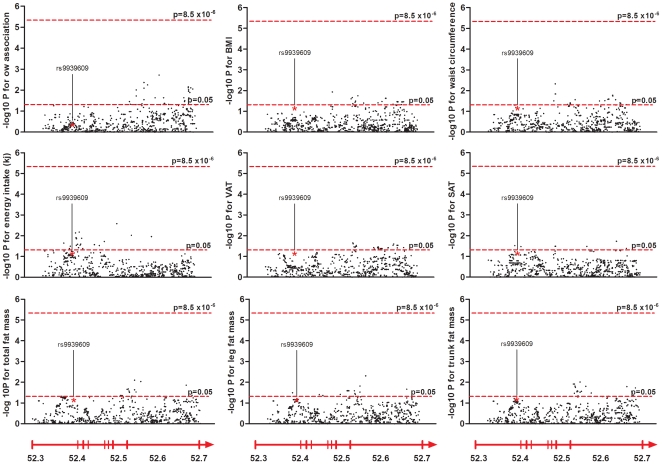
-log10 p-values for the single marker regression analysis of the studied phenotypes. Each dot represents a SNP (MAF >0.01). The dashed lines indicate significance thresholds; the lower one indicate unadjusted significance level (p<0.05) and the upper indicate the significance level after Bonferroni correction (p<8.5×10^−6^). Below each column the chromosomal position is shown and a schematic representation of the *FTO* gene indicated as an arrow. The previously associated variant, rs9939609, is marked with an asterisk. Abbreviations: ow, overweight, BMI, body mass index, VAT, visceral adipose tissue, SAT, subcutaneous adipose tissue.

Men and women were analyzed together since no significant gender difference for any of the studied variants in relation to anthropometric measurements and energy intake was detected (p>0.05 for all comparisons). Before controlling for multiple comparisons, 28 SNPs (not including rs9939609), all located in intron 8, were significantly linked (i.e. significance threshold: p<0.05) to overweight in both elderly men and women (Figure 1). Thirteen minor alleles of these SNPs were associated with increased susceptibility to develop overweight while the minor alleles of the remaining SNPs exerted an opposite effect. After Bonferroni correction, none of these 28 SNPs remained significant (p>8.5×10^−6^ for all comparisons).

For the previously studied rs9939609, the phenotypic level for carriers of the minor allele did not differ from carriers of the major allele for any of the studied phenotypes (p>0.05 for all phenotypes). Further analyses revealed that after correcting for multiple testing none of the SNPs were significantly associated (significance threshold: p<8.5×10^−6^) with BMI, waist circumference, total fat mass, trunk fat mass, leg fat mass, VAT, SAT, or daily energy intake (p>0.003 for all comparisons, Figure 1). Permutation testing also failed to identify significant association between SNPs and the studied phenotypes. The majority of SNPs with the highest rank (p<0.05) for BMI, waist circumference, total fat mass, trunk fat mass, leg fat mass, VAT and SAT were all located in intron 8. For energy intake the majority of SNPs were located in intron 1.

### Haplotype structure and LD evaluation

**Figure 2:**
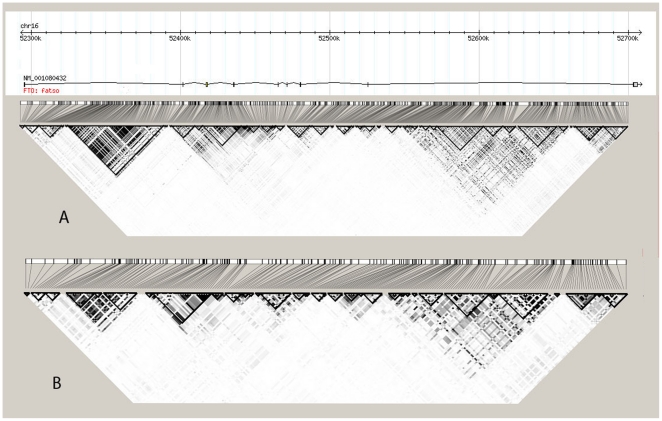
*FTO* gene structure, and LD pattern between all pairs of genotyped SNPs. The black triangles indicate haplotype blocks defined by confidence interval according to Gabriel et al. (Gabriel et al.). The diamonds represent the pair-wise linkage disequilibrium (LD) indicated as r^2^ (black r^2^>0.8, dark gray 0.5–0.8, moderate gray 0.4–0.6, light gray 0.2–0.4 and white r^2^<0.2). Each line represents a SNP. **A)** LD pattern and haplotype blocks based on the PIVUS data **B)** LD pattern and haplotype blocks based on HapMap data (version 3, release 27 and CEU+TSI). Only genotyped markers and haplotypes with a frequency >1% are included. The Haplotype analysis was performed using Haploview 4.1.

We constructed a LD map over the 733 genotyped SNPs, 184 being tagSNPs, (MAF >0.01) using Haploview according to [30] based on our 985 subjects. Figure 2 A depicts the gene structure, and the LD pattern between all pairs of SNPs indicated as r^2^ as well as the haplotype blocks. The haplotype structure based on the PIVUS samples comprised in total 44 haplotype blocks (Table S1) and 242 haplotypes (MAF >0.01) compared to the 29 blocks generated from 262 SNPs (MAF 0.01) and 112 subjects available in HapMap (Figure 2 B). The size of these blocks ranged from 33 bp to 42 kb. The largest, the 42 kb region, covered parts of intron 1 and encompassed 74 markers and 11 haplotypes. The strongest LD was seen in the first four introns (Figure 2 A) but the majority of the markers showed a relatively weak LD with 78% having an r^2^ between 1–25%.

### Multi-marker analysis

**Figure 3:**
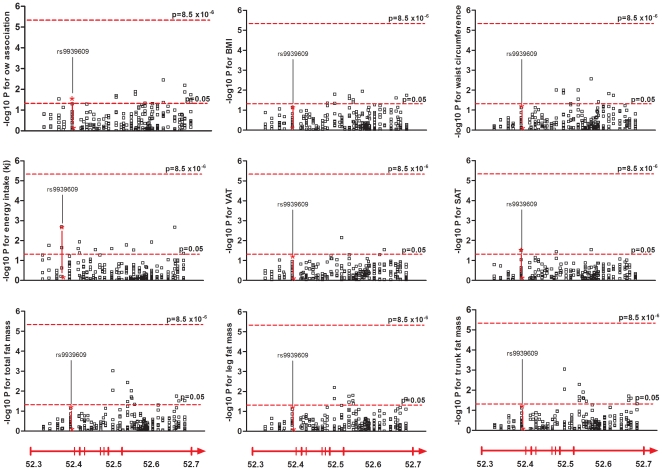
-log10 p-values for the haplotype analysis of the studied phenotypes. Each square represents a haplotype (MAF >0.01). The dashed lines indicate significance thresholds; the lower one indicate unadjusted significance level (p<0.05) and the upper indicate the significance level after Bonferroni correction (p<8.5×10^−6^). Below each column the chromosomal position is shown and a schematic representation of the FTO gene indicated as an arrow. Abbreviations: ow, overweight, BMI, body mass index, VAT, visceral adipose tissue, SAT, subcutaneous adipose tissue.

Haplotype analyses use the local LD structure of a candidate region and can be more informative than single marker analyses when causative variants are not directly genotyped. Based on the LD pattern constructed from our subjects we performed multi-marker analyses for overweight susceptibility and for the same phenotypes as for the single-marker analyses. 14 haplotypes in eight different blocks showed unadjusted association with overweight (Figure 3). Out of these, 7 haplotypes were shown to increase the susceptibility to overweight and 7 were shown to be protective. One of the haplotypes was located in intron 1, three in intron 6 and the remaining ten haplotypes were located in intron 8. Furthermore, in an unadjusted analysis, 53 haplotypes showed associations with fat mass and/or energy intake (p<0.05) (Figure 3). Subjects that were carriers of three of these haplotypes had increased levels of five or six of the studied phenotypes, compared to carriers of the other haplotypes in the same block. These were haplotypes in block 18, 22 and 44, located in intron 7 and intron 8. Subjects that were carriers of one haplotype in block 6, located in intron 1, which include rs9939609, only showed increased energy intake and amount of SAT compared to the subjects having the other haplotypes. However, after correcting for multiple testing and permutation testing with 10 000 permutations no haplotype was associated with any of the studied phenotypes (p>8.5×10^−6^ for all comparisons) (Figure 3).

## Discussion

In the present study, over 700 SNPs located in the *FTO* gene were genotyped in both elderly men and women at the age of 70 years. Neither a single SNP, such as rs9939609, nor a combination of SNPs was significantly linked to the level of overweight, BMI, body composition, waist circumference, or daily energy intake. These results hold both among men and women and indicate that the effect of *FTO* on body composition appears to be weakened with age. In a previous study of elderly men, the *FTO* rs9939609 minor allele was neither associated with obesity or BMI [16]. Our data also corroborate with the results presented by Hardy et al. [33] who show only a weak association between *FTO* and BMI at the age of 50 years.

BMI has been the most widely used measurement in the majority of studies between obesity and genetic variants in *FTO* [34], most likely due to its simple acquisition and calculation. BMI is a convenient surrogate for total fat mass; however, it offers no detailed insight into the regional body fat composition or distribution. Age-related changes in body composition include redistribution and increase of fat tissue, especially visceral fat, as well as a decrease in skeletal muscle and bone mass [17], [35]. Repositioning of fat and decreased muscle bulk may thus affect the validity of anthropometric measurements such as BMI in determining body composition [35]. Therefore, it is of great importance to administer more precise measurements of body adiposity rather than BMI in order to examine the effect of *FTO* in elderly humans. Total body composition measured by dual energy X-ray absorptiometry (DXA) offers good precision [36] and magnetic resonance imaging (MRI) enables the separate quantification of both visceral (VAT) and subcutaneous adipose tissue (SAT) [37]. Both methods are considered to be more clinically valuable than BMI in terms of assessing the individual risk for obesity-related morbidities in humans [38], [39], [40]. Therefore, in the present study, the regional assessment of body composition by DXA and MRI, together with BMI and waist circumference, allowed us to shed more light on the link between *FTO* and adiposity in elderly humans.

So far, it is unclear how *FTO* increases the susceptibility to develop overweight and obesity and whether this association is of correlative or causative origin. For instance, it could be due to an association between SNPs and variants elsewhere in the gene or control elements of other genes. However, there are data suggesting that the common variants in the *FTO* gene may increase body weight by influencing food intake [41], [42]. Carriers of at least one copy of this SNP more frequently choose energy-dense, palatable foods [14] and show signs of reduced satiety perception [15]. In the present study, however, the daily energy intake of elderly people, assessed by means of a seven-day diary was not significantly linked to SNPs in the *FTO* gene.

SNPs previously associated with BMI, lie within a 42 kb LD block encompassing parts of the first intron of the gene [1]. Only a few studies have analyzed variants outside of this region. Tönjes et al. [22] performed a genome-wide association study in the Sorbian population and detected an independent *FTO* signal, mapping to a region covering introns 2 and 3, about 40–60 kb away from the originally reported SNPs (rs9939609, rs1421085, and rs17817449). The strongest effect on BMI was therein associated with rs17818902 in intron 3 [22]. Adeyemo et al. [43] showed that several SNPs intron 8, in addition to SNPs in intron, were significantly associated with BMI, waist circumference, and percent fat mass. Furthermore, we previously screened exons 3 and 4 including exon-intron boundaries among obese children and adolescents and identified intronic variants in intron 4, only about 7 kb away from rs17818902, that caused increased fasting serum insulin levels and increased degree of insulin resistance [21]. This suggests that the genomic region of *FTO* might harbor multiple variants that influence susceptibility to develop obesity or related traits and indicate the importance of studying a broader range of SNPs in a wider region. While single marker analyses only consider a single marker at a time, methods of haplotype analysis use information from nearby markers and tend to have enhanced power for detecting complex disease genes [23], [44], [45]. More importantly, haplotype methods use the LD structure of the region and are more informative than single marker analyses when the causative variant has not been selected for genotyping. Also, haplotypes has been shown to, in general, be conserved across human populations [46], [47] and certain genes have a much conserved haplotype [48]. Our very dense haplotype map over the *FTO* gene could therefore be translatable to other populations and be of general interest. The haplotype analysis enabled us to limit the search for the causal region/variant and to search for additional regions. We observed high ranks for some of the haplotypes in intron 8 as for the single marker analyses, supporting the possibility of additional linked variants in this region which may have a strong effect among younger men and women. However, no single SNP or haplotype were near the gene-wide significance level among these elderly subjects. In contrast, a number of earlier positive studies for the association between the *FTO* gene and obesity have been done in children and adolescents [4], [5], [6], [7], [8], [9], [10], [11]. Furthermore, results from twin research on adults indicate that the genetic influence on obesity and fat mass decreases with increasing age [49], [50], an observation that both can be explained by a strengthened lifestyle influence at old age but also partially by survival bias associated with the *FTO* gene [51].

The strengths of this study are threefold: it is conducted in homogenous, community-based cohort of men and women aged 70 years, it incorporates an extensive number of accurately and precisely determined body weight composition measures, and includes a haplotype analysis rather than narrowly focusing on one or a small number of major SNPs. However, the single-marker analysis is limited by its lack of generalisability to other ethnicities, in that the participants were predominantly Caucasian. It is furthermore well known that self-reported food records are not very reliable and that underreporting is a frequent problem, particular in overweight and obese subjects. A validation study in adults on food intake obtained with two different methods; weighted food records and the precoded food records used in this study indicated some differences in the amount of energy consumed of some nutrients and especially in intake of proteins and fat, which was higher in the precoded food records [28]. This show that underreporting is not a problem in a general population for the precoded record but under- or overreporting in obese versus normal-weight subjects has not been studied. However, the overall result show that the average daily intake of energy obtained with the precoded food records do not differ from weighted food records [28]. Moreover, since our power was limited, studies with the same phenotypes available but with greater sample sizes are required in order to replicate these findings.

### Conclusion

Common variants in the *FTO* gene are linked to obesity in childhood and young adulthood [1]. In the present cross-sectional study, we were not able to replicate these findings inasmuch as neither SNPs nor haplotypes in the *FTO* gene were associated with anthropometric measurements including body fat or daily energy intake obtained by a seven-day food protocol. These results, in combination with previous findings, provide additional evidence that the effect of *FTO* is weakened among elderly.

## Supporting Information

Table S1 Haplotype blocks constructed from 733 SNPs in *FTO*.
(DOCX)
