# Supplementary material for: Detailed Analysis of Variants in FTO in Association with Body Composition in a Cohort of 70-Year-Olds Suggests a Weakened Effect among Elderly
Source: PLoS One. 2011 May 27;6(5):e20158. doi: 10.1371/journal.pone.0020158 (PMC3103532; doi:10.1371/journal.pone.0020158)
Supplement: Table S1 — Haplotype blocks constructed from 733 SNPs in FTO. (DOCX) [file pone.0020158.s001.docx]

| **Supplementary table 1:** Haplotype blocks constructed from 733 SNPs in *FTO*. | | | | | | | | |
| --- | --- | --- | --- | --- | --- | --- | --- | --- |
| **Block** | **Size (bp)** | **Start** | **End** | **Markers** |  |  |  |  |
| 1 | 8211 | 52299427 | 52307638 | Chr16:52299427 | Chr16:52304376 | rs4389136 | rs12447481 | Chr16:52306950 |
|  |  |  |  | rs12445162 |  |  |  |  |
| 2 | 16437 | 52312647 | 52329084 | rs7205986 | rs7206010 | rs4386132 | rs4280233 | rs7203521 |
|  |  |  |  | rs6499640 | rs8048396 | rs16952482 | rs11861870 | rs17217144 |
|  |  |  |  | rs11643744 | rs7184874 | rs13333228 |  |  |
| 3 | 21783 | 52330548 | 52352331 | rs4396532 | Chr16:52332719 | rs62048372 | rs2388256 | rs7186637 |
|  |  |  |  | Chr16:52346350 | rs1108103 | rs2892469 | rs1861868 | rs1075439 |
|  |  |  |  | rs1075440 | rs1077128 | rs7186521 | rs7191566 | Chr16:52351315 |
|  |  |  |  | rs17525605 | rs8056666 |  |  |  |
| 4 | 33 | 52353137 | 52353170 | rs13334933 | rs61507259 | Chr16:52353170 |  |  |
| 5 | 47 | 52355019 | 52355066 | rs6499643 | rs4784323 |  |  |  |
| 6 | 43269 | 52355409 | 52398678 | rs7206790 | rs8047395 | rs8047587 | rs9937053 | rs9937354 |
|  |  |  |  | rs9928094 | rs9930333 | rs9930397 | rs9939973 | rs9940646 |
|  |  |  |  | rs9940128 | rs1421085 | rs9923147 | rs9923544 | rs11642015 |
|  |  |  |  | rs8055197 | rs1558901 | rs62048402 | rs1558902 | rs1861866 |
|  |  |  |  | rs10852521 | rs7187250 | rs7193144 | rs8063057 | rs16945088 |
|  |  |  |  | rs8057044 | rs17817449 | rs8063946 | rs9972653 | rs11075987 |
|  |  |  |  | rs17817497 | rs8050136 | rs4783819 | rs8051591 | rs9935401 |
|  |  |  |  | rs9933509 | rs3751812 | rs3751813 | rs3751814 | rs9931900 |
|  |  |  |  | rs9936385 | rs11075989 | rs11075990 | rs11075991 | rs11075992 |
|  |  |  |  | rs9926289 | rs9939609 | rs17817712 | rs7206410 | rs7206629 |
|  |  |  |  | rs7202116 | rs7202296 | rs7201850 | rs7202296 | Chr16:52379684 |
|  |  |  |  | rs7185735 | rs62033406 | rs9941349 | rs7187961 | rs10468280 |
|  |  |  |  | rs62033408 | rs17817964 | rs7190492 | rs9930506 | rs9933040 |
|  |  |  |  | rs9922708 | Chr16:52388855 | rs9922619 | rs4783821 | rs11075993 |
|  |  |  |  | Chr16:52394843 | rs62033415 | rs8044769 | rs12149182 |  |
| 7 | 1671 | 52400409 | 52402080 | rs12149832 | rs17218700 |  |  |  |
| 8 | 3074 | 52402988 | 52406062 | rs11642841 | rs1861867 |  |  |  |
| 9 | 35 | 52408369 | 52408404 | rs9939811 | rs56057753 |  |  |  |
| 10 | 14983 | 52408805 | 52423788 | rs9972717 | rs8054859 | rs8054908 | rs11075995 | rs17219084 |
|  |  |  |  | rs62033422 | rs62033423 | rs56390114 | rs11075996 | rs56897002 |
|  |  |  |  | rs10852522 | rs7190842 | rs7195279 | rs3848299 | rs8058930 |
|  |  |  |  | rs8061518 | rs6499648 | Chr16:52419044 | rs7184573 | rs9923295 |
|  |  |  |  | rs59047767 | rs10521307 | rs9302652 | rs9934528 |  |
| 11 | 2811 | 52424268 | 52427079 | rs62033429 | rs2388405 | rs62033431 | rs16952577 | rs7201255 |
|  |  |  |  | rs12596296 | rs61429622 |  |  |  |
| 12 | 14674 | 52427841 | 52442515 | rs12596393 | rs17818824 | rs17818866 | rs17818890 | rs61222151 |
|  |  |  |  | rs17818997 | rs17819063 | rs6499651 | rs6499652 | rs7190053 |
|  |  |  |  | rs17219983 | rs16952581 | Chr16:52432470 | rs2111115 | rs8053367 |
|  |  |  |  | rs8055484 | rs7197885 | rs7203883 | rs7205213 | rs2388406 |
|  |  |  |  | rs6499653 | rs62033438 | rs62033439 | Chr16:52436593 | Chr16:52436615 |
|  |  |  |  | rs52436971 | rs61471078 | rs55798112 | rs55835063 | rs55844219 |
|  |  |  |  | rs12447581 | Chr16:52439466 | rs8061228 | rs12448529 | rs11075999 |
|  |  |  |  | rs1344500 | rs2111114 | rs2388409 | rs12596457 |  |
| 13 | 1949 | 52443019 | 52444968 | rs11076001 | rs9934978 | rs9934979 | rs13337496 | rs13337587 |
|  |  |  |  | rs13337591 | rs13337898 |  |  |  |
| 14 | 7760 | 52445239 | 52452999 | rs12597422 | rs7202620 | rs9933461 | rs9938445 | rs9936319 |
|  |  |  |  | Chr16:52447786 | Chr16:52449408 | rs8063241 | rs11076003 |  |
| 15 | 12281 | 52455415 | 52467696 | rs28551130 | rs2024471 | Chr16:52459394 | rs7197983 | rs8060649 |
|  |  |  |  | rs6499654 | rs7188378 | rs9673307 | rs10521304 | rs10521303 |
|  |  |  |  | rs4784328 |  |  |  |  |
| 16 | 2205 | 52467762 | 52469967 | rs4784329 | rs7191718 | rs1362571 | rs8061239 | rs9933461 |
| 17 | 8950 | 52470388 | 52479338 | rs9931209 | Chr16:52471803 | rs1558756 | rs9934504 | Chr16:52478108 |
|  |  |  |  | rs1558755 | rs56335873 | rs16952623 |  |  |
| 18 | 19410 | 52485099 | 52504509 | rs1344498 | rs12149433 | rs9926180 | Chr16:52486642 | rs2111113 |
|  |  |  |  | rs13335343 | Chr16:52489404 | rs2111112 | rs9937234 | rs10852525 |
|  |  |  |  | rs9929152 | rs56379708 | rs11076010 | rs8056040 | rs4784330 |
|  |  |  |  | rs12935710 | rs34533783 | rs12708942 | rs7188162 | rs12929364 |
| 19 | 308 | 52508854 | 52509162 | rs7197167 | rs4783824 |  |  |  |
| 20 | 8383 | 52510620 | 52519003 | rs12232391 | rs8053966 | rs17222911 | rs7194336 | rs17821714 |
|  |  |  |  | rs16952649 | rs7201878 | rs11076011 | rs16952657 | rs7204611 |
|  |  |  |  | rs11860076 | rs35013387 | rs12918495 | rs12933343 | rs4783826 |
|  |  |  |  | rs12919488 |  |  |  |  |
| 21 | 1099 | 52522465 | 52523564 | Chr16:52522465 | rs9924072 |  |  |  |
| 22 | 14388 | 52528386 | 52542774 | rs2024470 | Chr16:52529572 | Chr16:52530881 | rs7205426 | Chr16:52531569 |
|  |  |  |  | rs10521302 | rs12933996 | Chr16:52536132 | rs12931934 | rs12919344 |
|  |  |  |  | rs9924877 | rs7202360 | rs2287141 | rs7203181 | rs35090620 |
|  |  |  |  | rs12925189 |  |  |  |  |
| 23 | 23 | 52546456 | 52546479 | rs6499656 | rs6499657 |  |  |  |
| 24 | 2695 | 52546608 | 52549303 | Chr16:52546608 | rs35109715 | Chr16:52546943 | rs7185301 | rs16952686 |
|  |  |  |  | rs7185479 | rs7191513 | rs35951481 |  |  |
| 25 | 11196 | 52549788 | 52560984 | rs57957459 | rs6499658 | rs1861555 | rs11643535 | rs12446047 |
|  |  |  |  | rs17823199 | rs17823223 | rs1344502 | rs7194907 |  |
| 26 | 390 | 52562600 | 52562990 | rs9932394 | rs35029771 | rs9932411 | rs7206456 |  |
| 27 | 1103 | 52565726 | 52566829 | chr16:52565726 | rs7192835 | rs9921255 |  |  |
| 28 | 9420 | 52567002 | 52576422 | rs8056299 | rs8057547 | rs9302654 | rs4784335 | rs28478013 |
|  |  |  |  | rs16952728 | rs17225435 | Chr16:52575602 | rs3038575 | rs16952730 |
| 29 | 53 | 52577134 | 52577187 | rs12325409 | rs12324955 |  |  |  |
| 30 | 2026 | 52578510 | 52580536 | rs8049235 | rs10550005 | rs4784336 | rs62035803 |  |
| 31 | 68 | 52581017 | 52581085 | rs9933107 | rs9933805 |  |  |  |
| 32 | 1032 | 52582673 | 52583705 | rs8056199 | rs8056502 | rs6499660 |  |  |
| 33 | 3583 | 52584182 | 52587765 | rs6499661 | rs7194830 | rs1420571 | rs12596210 | rs12596862 |
|  |  |  |  | rs11864881 | rs11076013 | rs17824224 |  |  |
| 34 | 1588 | 52588988 | 52590576 | rs35702217 | rs8046658 | rs10595871 | rs8062658 | rs12385988 |
|  |  |  |  | rs8047473 | rs11646488 |  |  |  |
| 35 | 3108 | 52590636 | 52593744 | rs7193938 | rs7199716 | rs1861356 | rs1861357 | rs7205987 |
|  |  |  |  | rs16952770 | rs13335453 |  |  |  |
| 36 | 23853 | 52593853 | 52617706 | rs7200972 | rs1079880 | rs16952777 | rs4784338 | rs9925908 |
|  |  |  |  | rs2388451 | rs7199363 | rs12931859 | rs4784339 | rs9922370 |
|  |  |  |  | rs1971037 | rs1541577 | rs1125337 | rs1125338 | rs8049988 |
|  |  |  |  | rs1125339 | rs8054364 | rs6499664 | rs16952808 | rs7201494 |
|  |  |  |  | Chr16:52600668 | rs12600130 | rs17226942 | rs1345390 | rs1861358 |
|  |  |  |  | rs2111116 | rs1861554 | rs1990685 | rs7196211 | rs6499667 |
|  |  |  |  | rs2080366 | rs2080450 | rs2080449 | rs10153154 | rs1075886 |
|  |  |  |  | rs6499669 | rs6499670 | rs10589141 | rs7186220 | rs1110490 |
|  |  |  |  | rs1107357 | rs35236578 | rs7194243 | rs4784346 | rs17825567 |
|  |  |  |  | rs7195994 |  |  |  |  |
| 37 | 5729 | 52617999 | 52623728 | rs2302675 | rs2302674 | rs7500983 | Chr16:52618911 | rs12443572 |
|  |  |  |  | rs7185938 | rs4784347 | rs4784347 | rs62034079 | rs1477093 |
|  |  |  |  | rs12051261 | rs12051239 | rs8049544 | rs13331869 | rs13339176 |
| 38 | 13960 | 52626708 | 52640668 | rs856976 | rs860713 | rs856979 | rs2689251 | rs856983 |
|  |  |  |  | rs3764307 | rs2192869 | rs13330831 | Chr16:52639635 | rs6499675 |
|  |  |  |  | rs2689249 | rs16952906 |  |  |  |
| 39 | 17072 | 52643195 | 52660267 | rs1078013 | rs1076467 | rs11076015 | rs2540781 | rs2689271 |
|  |  |  |  | rs8062891 | rs940214 | rs1102175 | rs58616327 | rs8049933 |
|  |  |  |  | rs856974 | rs59643213 | rs62034112 | rs62034113 | rs1558687 |
|  |  |  |  | rs2689246 | rs2003583 | rs1108086 | rs1076471 | rs2075204 |
|  |  |  |  | rs1420318 |  |  |  |  |
| 40 | 5550 | 52662960 | 52668510 | rs28526719 | rs2540766 | rs2540768 | rs2540769 | rs2665275 |
|  |  |  |  | rs7204690 | rs16952993 |  |  |  |
| 41 | 1136 | 52669972 | 52671108 | rs62034118 | rs7206012 | rs7199185 |  |  |
| 42 | 267 | 52671763 | 52672030 | rs11646290 | rs708262 |  |  |  |
| 43 | 12184 | 52672325 | 52684509 | rs16953002 | rs697771 | rs708259 | rs708258 | rs1610275 |
|  |  |  |  | rs12596638 | rs17236232 | rs12599672 | rs62034120 | rs3928987 |
|  |  |  |  | rs62034121 | rs1876941 | rs918029 | rs918030 | rs918032 |
|  |  |  |  | rs1008400 | rs10492872 | rs11076017 | rs9937121 | rs11646512 |
|  |  |  |  | rs697769 | Chr16:52679663 | rs708256 | rs708255 | rs708254 |
|  |  |  |  | rs11863548 | rs708252 | rs2689253 | rs11076018 | rs2665272 |
|  |  |  |  | rs61121127 |  |  |  |  |
| 44 | 19524 | 52685184 | 52704708 | rs12932373 | rs2665271 | rs2689248 | rs17833492 | rs17236708 |
|  |  |  |  | rs16953047 | rs2540772 | rs1071501 | rs62034139 | rs62034140 |
|  |  |  |  | rs16953048 | rs718388 | rs741300 | rs12927155 | Chr16:52691601 |
|  |  |  |  | rs12445828 | rs11076020 | rs28579391 | rs2689264 | rs2689269 |
|  |  |  |  | rs10163409 | rs10163246 | rs10163276 | rs3885808 | rs17236863 |
|  |  |  |  | Chr16:52701116 | rs708278 | rs5816925 |  |  |
| The start and end position of the blocks is listed as well as the size of the block as base pair. All SNPs included in the haplotype blocks are listed with the reference SNP id. For those SNPs without a rs-id, the chromosomal position is used as id. | | | | | | | | |
